# Bonit Coating Leads to Macroscopic Bone Ingrowth at 8 Weeks After Primary Total Hip Arthroplasty

**DOI:** 10.1016/j.artd.2022.06.004

**Published:** 2022-07-19

**Authors:** Moritz Wagner, Alexander Brunner, Gerhard Kaufmann, Dietmar Dammerer, Paul Nardelli, Erwin Schwaighofer

**Affiliations:** a Department of Orthopedic Surgery, District Hospital St. Johann, St. Johann, Austria; b OFZ Innsbruck, Innsbruck, Austria; c Department of Orthopedic Surgery, Krems Donau Universität, Krems, Austria; d Medizinische Universität Innsbruck, Innsbruck, Austria; e Landesklinikum Scheibbs, Scheibbs, Austria

**Keywords:** Arthroplasty, Bonit, Coating, Ingrowth, Osseointegration

## Abstract

Primary total hip arthroplasty with cementless stems has numerous advantages over cemented total hip arthroplasty in patients with good bone quality. To enhance osseointegration with ingrowth into the implant, various surface treatments have been proposed. Multiple biomechanical studies in animals have shown that bioactive coatings enhance osseointegration and increase construct stability. Bony ingrowth in humans can only be assessed in rare instances of periprosthetic femoral fractures. In this case report, we describe the findings after a periprosthetic fracture mandating stem exchange in a patient who experienced a fall 8 weeks after implantation. The retrieved proximal Bonit (DOT GmbH, Rostock, Germany) coated stem showed substantial macroscopically visible trabecular bone. This finding supports results from animal studies that showed enhanced metaphyseal bone ingrowth with Bonit coating of implants.

## Introduction

Primary total hip arthroplasty (THA) with cementless stems has numerous advantages over cemented THA in patients with good bone quality. However, initial construct stability is lower with press-fit stems than with cemented stems [1]. Overall construct stability improves much more over time through the process of osseointegration [2]. Whether restricted weight-bearing in the initial weeks after THA has beneficial effects regarding osseointegration is unclear. Compliance toward restricted weight-bearing is low in patients who have undergone arthroplasty [3]. Weight-bearing as tolerated is state of the art, and patient expectations from initial construct stability are high [4]. The time necessary for complete osseointegration is known from animal studies but can only be estimated for human application. To enhance osseointegration, extensive research has been conducted on the ideal properties for orthopedic implants. The implant surface characteristics that are most important include pore size, pore density, and presence of a bioactive coating on the implant surface [5]. The most frequently used technique to roughen titanium (Ti-6Al-4V) implants and create a porous surface is titanium plasma spray (TPS), while the less frequently used methods are corundum blasting and glass-bead blasting [6,7]. Implants with bioactive coatings have been shown to be superior to uncoated ones [8]. Hydroxyapatite (HA) was one of the first bioactive coatings and has been used successfully without additional compounds; it has been used with a thickness of 50-200 μm. Its successor, calcium-phosphate HA (Bonit; DOT GmbH, Rostock, Germany), also known as third-generation HA, is applied at a thickness of only 10-30 μm. Bonit contains more than 70% of calcium phosphate (brushite) mixed with less than 30% of HA; it is used for titanium plasma-sprayed titanium implants. Bonit has a higher capillary attraction than conventional HA, resulting in complete resorption of the coating over time. Its biological properties have been compared to those of conventional HA in a rabbit model, and Bonit showed steadily increasing osseointegration with a mechanically more resistible bone-implant interface [9].

The in vivo effects of Bonit on stem osseointegration in humans can only be assessed in rare cases where surgery with stem exchange is necessary. Stem exchange without disturbed osseointegration is mainly necessary in cases of periprosthetic fractures or implant malpositioning. We describe a case of a patient who sustained a Vancouver B2 periprosthetic fracture 8 weeks after primary THA for which stem exchange was necessary. We found strong osseointegration with visible trabecular bone ingrowth at the Bonit-coated part of the stem.

### Case history

**Figure 1:**
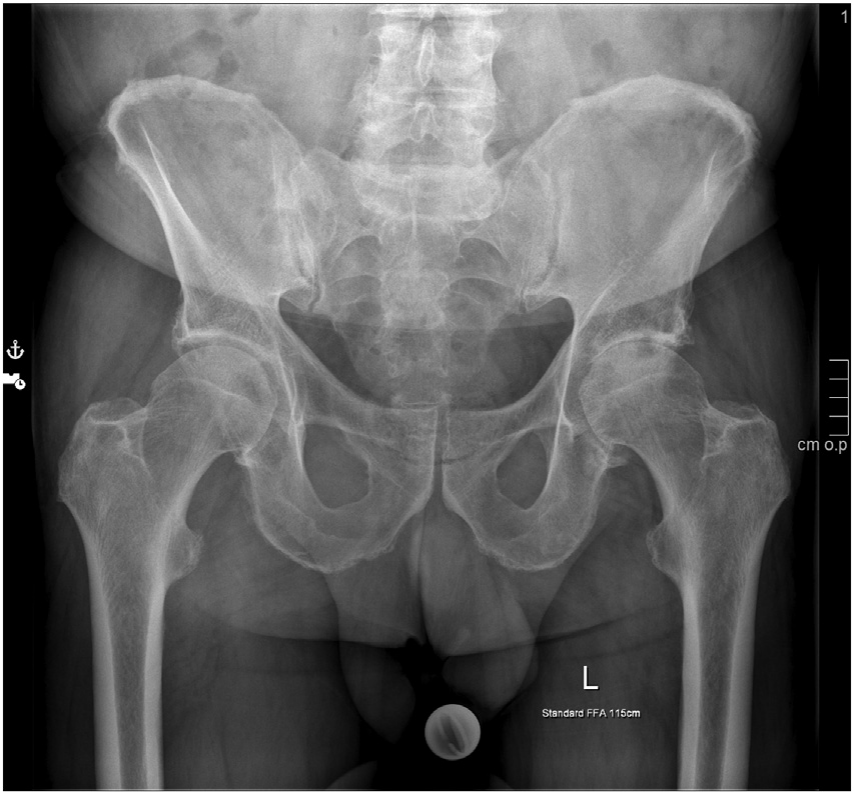
Preoperative radiograph of the patient with hip osteoarthritis (Kellgren-Lawrence Stage 3).

Written informed consent for publication has been obtained from the patient. A 74-year-old man had been admitted to a regional orthopedic clinic for the management of end-stage hip osteoarthritis (Fig. 1). The decision was made to perform primary THA using the direct anterior approach. The patient was a nonsmoker with no relevant comorbidities and an American Society of Anesthesiologists-2 physical status. The patient underwent implantation of a cementless acetabular cup (Siocon; Falcon Medical, Mödling, Austria) with a diameter of 60 mm involving a fourth-generation ceramic metal-backed liner (Biolox delta; CeramTec GmbH, Plochingen, Germany).

A cementless stem (ProMIS; Falcon Medical, Mödling, Austria) of size 5 and a 36-mm ceramic head (+4 mm) were inserted into the proximal femur. This stem has a double taper and a reduced lateral shoulder for facilitated insertion with the direct anterior approach. The femoral canal is prepared with broaches of increasing size. This stem was treated with TPS and bioactive Bonit coating in the proximal metaphyseal region, corresponding to Gruen zones 1 and 7 [10], to allow for solid osseointegration in the metaphyseal region. The distal area of the stem was only roughened with TPS to allow for stable diaphyseal press-fit fixation, unobstructed by debris from the Bonit coating during stem insertion.

**Figure 2:**
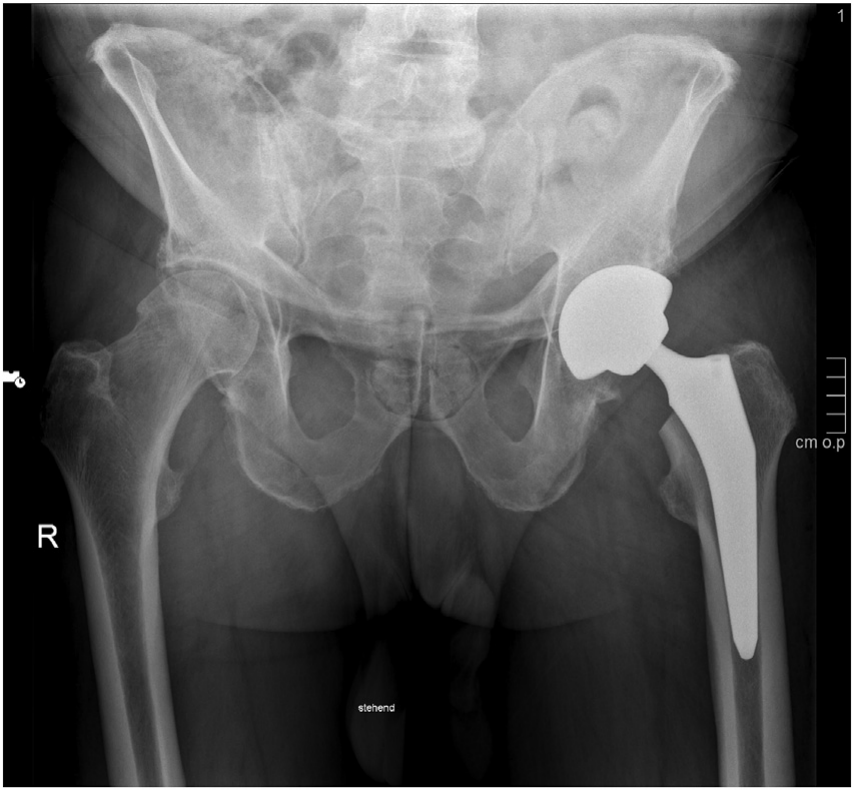
Postoperative radiograph obtained after the procedure.

Radiographs obtained on the day after surgery showed correct implant positioning, and the cup and stem were positioned according to the preoperative template (Fig. 2). The patient was soon ambulatory, showed no complications during the in-hospital stay, and could be discharged after 4 days.

**Figure 3:**
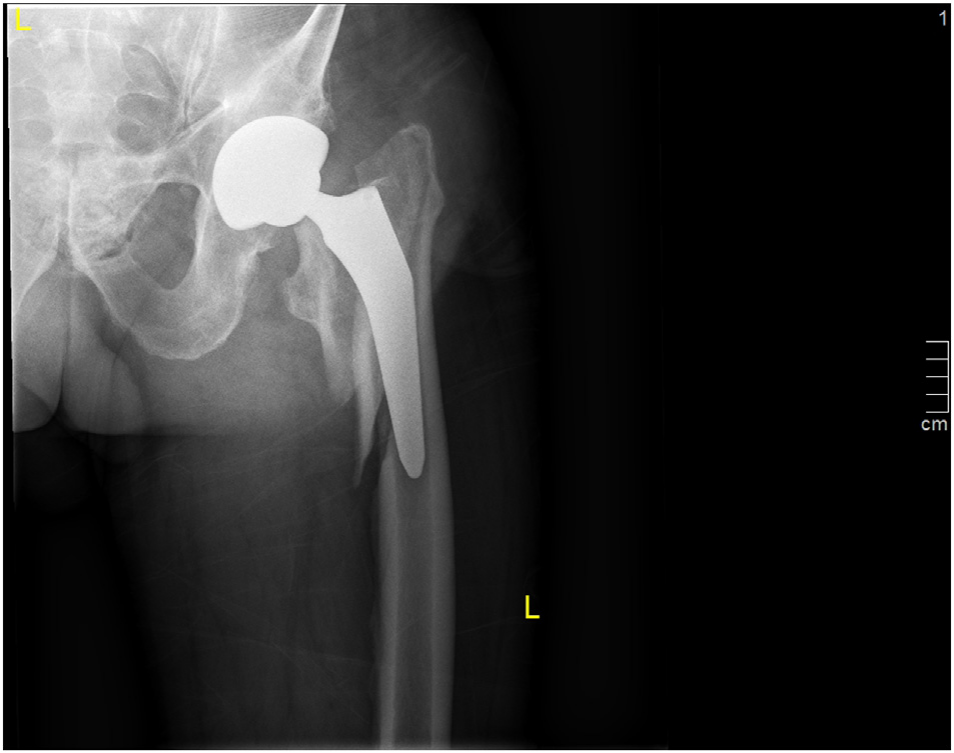
Vancouver B2 periprosthetic fracture after a fall 8 weeks after surgery.

The first clinical follow-up assessment after 2 weeks showed unremarkable findings. The patient had no complaints and was ambulatory without help. The wounds had healed, and radiography showed adequate implant positioning with no evidence of implant migration. Eight weeks later, on the way to his musculoskeletal rehabilitation facility, the patient stumbled and fell on his left hip. A radiograph obtained subsequently showed a periprosthetic femoral fracture with a loose stem (Fig. 3). Revision THA was performed, the mechanically resistible stem was removed, and a Zweymüller Alloclassic revision stem size 7 (Zimmer Biomet Holdings, Warsaw, IN) was implanted with a 36-mm ceramic head (+0 mm). The reduced fracture was secured using 3 Gundolf compression cerclages (ImplanTec GmbH, Mödling, Austria). No complications were observed after revision surgery.

**Figure 4:**
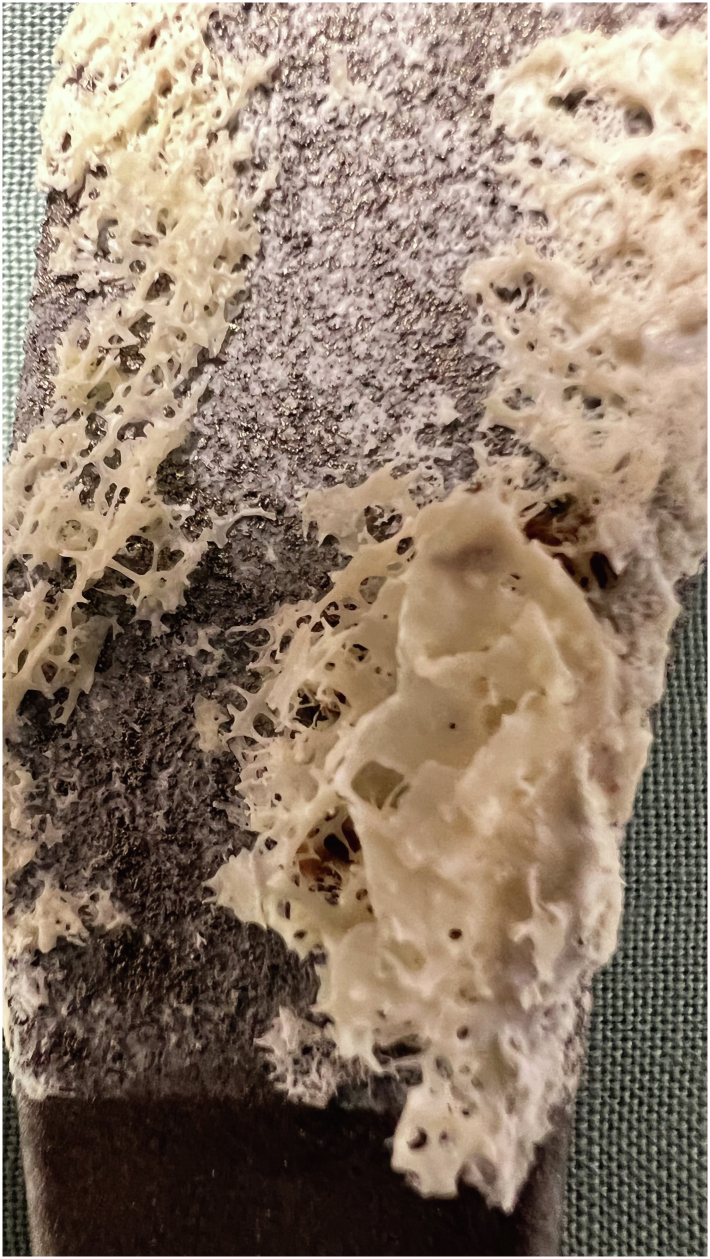
Visible osseointegration at the Bonit-coated proximal part, with no osseointegration observed on the distal part of the stem.

**Figure 5:**
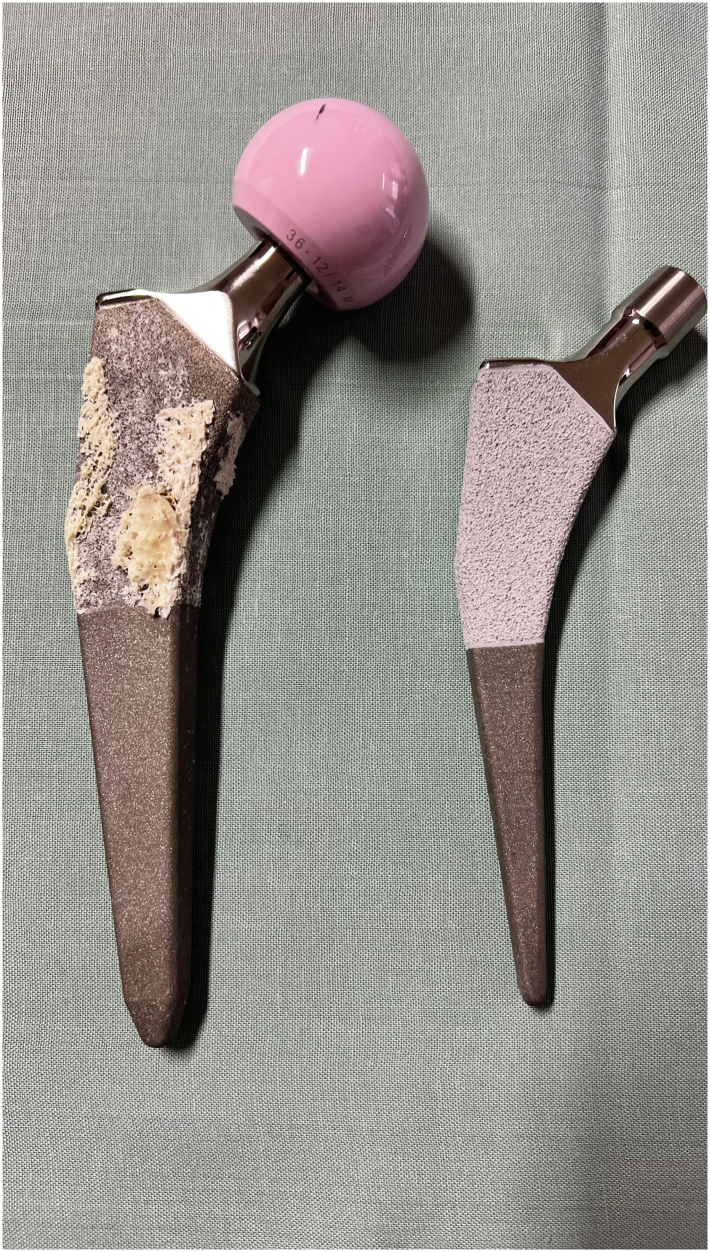
Osseointegration with trabecular structure visible on the Bonit-coated part of the stem.

The explanted ProMIS stem was cleaned of blood clots and loose debris. Visual inspection showed a substantial bone ingrowth in the proximal metaphyseal region of the stem, corresponding to Gruen zones 1 and 7, where it was previously coated with calcium phosphate. The trabecular structure of the bone had adhered strongly to the implant surface and was clearly visible on inspection (Fig. 4). The distal part of the stem, corresponding to Gruen zones 2-6, showed no signs of trabecular bone ingrowth (Fig. 5).

## Discussion

This case graphically illustrates the properties of Bonit coating in a proximally coated stem. The long-term clinical outcomes for patients with these implants have been reported to be excellent [11]. Although numerous studies have evaluated the role of the bioactive coating on osseointegration, its clinical implications can only be depicted in rare cases that mandate stem exchange, such as this one.

Radiostereometric studies have been successfully used in the past to measure implant migration [12] and have also been used to evaluate the effects of the stem coating on implant migration. In one such radiostereometric study, Paul van der Voort [13] found no differences in long-term stem migration among HA, fluorapatite, and no coating.

Biomechanical animal studies have also been performed to evaluate the effects of the implant coating on construct stability and osseointegration. The results of these studies supported the use of HA-coated stems and showed faster, stronger, and more durable osseointegration than that obtained with conventional stems in canine animal models [14,15]. Clinical retrospective studies also support the use of bioactive coating. HA has been shown to increase clinical long-term survival and is therefore superior to no coating [8].

In vivo osseointegration of stems can only be observed in cases of revision surgery. We are aware of 1 case series of 4 patients who underwent revision procedures at different time points due to dislocation, periprosthetic fractures, and infection. These patients underwent primary THA with a stem very similar to this case, since both were proximally coated with Bonit. As in our case, these stems showed rapid and extensive osseointegration in the coated region [16]. The limitations of this case report include the lack of histological analysis and the lack of scanning electron microscope analysis of the explanted stem. However, macroscopic trabecular bone ingrowth was clearly visible on the implant, and it was mechanically resistible and firmly attached. The strength of this case report is the fact that only very few cases of periprosthetic fracture that mandate stem exchange of Bonit-coated implants early after implantation have been reported in the literature. Only in those rare cases, bony ingrowth of implants can be reliably observed.

### Summary

Coating with Bonit has been shown to be safe and effective in enhancing osseointegration [14,17,18]. Ingrowth of the trabecular bone in the metaphyseal region increases early construct stability. Bioactive coatings add to the circumference of the stem and are therefore not ideal in the diaphyseal region where the stem should be tight-fitting in the cancellous canal [16]. The use of Bonit seems to enhance femoral stem osseointegration in the metaphyseal region.

**Key Points**

• A retrieved Bonit-coated implant showed trabecular bone ingrowth 8 weeks after implantation.
• Bonit coating is thinner and more quickly absorbed than conventional hydroxyapatite coatings.
• Bonit seems to enhance femoral stem osseointegration in the metaphyseal region.

## Conflict of interest

The authors declare there are no conflicts of interest.

For full disclosure statements refer to https://doi.org/10.1016/j.artd.2022.06.004.

## Informed patient consent

The authors confirm that informed consent has been obtained from the involved patient or if appropriate from the parent, guardian, power of attorney of the involved patient; and, they have given approval for this information to be published in this article.

## Appendix A Supplementary Data

Conflict of Interest Statement for Brunner

Conflict of Interest Statement for Dammerer

Conflict of Interest Statement for Kaufmann

Conflict of Interest Statement for Nardelli

Conflict of Interest Statement for Schwaighofer

Conflict of Interest Statement for Wagner
