# Supplementary material for: Bonit Coating Leads to Macroscopic Bone Ingrowth at 8 Weeks After Primary Total Hip Arthroplasty
Source: Arthroplast Today. 2022 Jul 19;16:203–6. doi: 10.1016/j.artd.2022.06.004 (PMC9304667; doi:10.1016/j.artd.2022.06.004)
Supplement: Conflict of Interest Statement for Kaufmann [file mmc3.pdf]

INDIVIDUAL CONFLICT OF INTEREST STATEMENT

American Association of Hip and Knee Surgeons

(Adopted from the American Academy of Orthopaedic Surgeons disclosure statement)

The following form **<u>must be filled out completely and submitted by each author (example, 6 authors, 6 forms). All items require a response. If there is no relevant disclosure for a given item, enter "*None.*"</u>**

Manuscript Title

1. Royalties from a company or supplier (The following conflicts were disclosed)

None.

2. Speakers bureau/paid presentations for a company or supplier (The following conflicts were disclosed)

None.

3A. Paid employee for a company or supplier (The following conflicts were disclosed)

None.

3B. Paid consultant for a company or supplier (The following conflicts were disclosed)

None.

3C. Unpaid consultants for a company or supplier (The following conflicts were disclosed)

None.

4. Stock or stock options in a company or supplier (The following conflicts were disclosed)

None.

5. Research support from a company or supplier as a Principal Investigator (The following conflicts were disclosed)

None.

6. Other financial or material support from a company or supplier (The following conflicts were disclosed)

None.

7. Royalties, financial or material support from publishers (The following conflicts were disclosed)

None.

8. Medical/Orthopaedic publications editorial/governing board (The following conflicts were disclosed)

None.

9. Board member/committee appointments for a society (The following conflicts were disclosed)

None.

<u>Each author must sign AND print or type his/her name, date and submit a separate form</u>

In addition, one BLINDED Conflict of Interest form (no author names used) should be submitted per manuscript with all author disclosures.

Kaufmann Gerhard | [signature] | 21.04.22

Author Name (Print or Type) | Author Signature | Date

Priv.-Doz. Dr. Gerhard Kaufmann
FA für Orthopädie und orthop. Chirurgie
Rheumaorthopädie, Sportmedizin,
Manuelle Medizin, Akupunktur
Innrain 2/6020 Innsbruck
Tel.: 0512/563900 Fax: 0512/563900-6
